# What factors explain the much higher diabetes prevalence in Russia compared with Norway? Major sex differences in the contribution of adiposity

**DOI:** 10.1136/bmjdrc-2020-002021

**Published:** 2021-03-04

**Authors:** Olena Iakunchykova, Maria Averina, Tom Wilsgaard, Sofia Malyutina, Alexander V Kudryavtsev, Sarah Cook, Sarah Wild, Anne Elise Eggen, Laila Arnesdatter Hopstock, David A Leon

**Affiliations:** 1 Department of Community Medicine, UiT The Arctic University of Norway, Tromso, Norway; 2 Department of Laboratory Medicine, University Hospital of North Norway, Tromso, Troms, Norway; 3 Research Institute of Internal and Preventive Medicine, Branch of Institute of Cytology and Genetics, Siberian Branch of the Russian Academy of Sciences, Novosibirsk, Russia; 4 Novosibirsk State Medical University, Russian Ministry of Health, Novosibirsk, Russia; 5 Department of Innovative Programs, Northern State Medical University, Arkhangelsk, Russia; 6 Faculty of Epidemiology and Population Health, London School of Hygiene & Tropical Medicine, London, UK; 7 Usher Institute, The University of Edinburgh, Edinburgh, UK; 8 Department of Non-communicable Disease Epidemiology, London School of Hygiene & Tropical Medicine, London, UK; 9 International Laboratory for Population and Health, National Research University Higher School of Economics, Moscow, Russia

**Keywords:** body mass index, C-reactive protein, diabetes mellitus, type 2, obesity

## Abstract

**Introduction:**

Compared with many other countries Russia has a high prevalence of diabetes in men and women. However, contrary to what is found in most other populations, the risk is greater among women than men. The reasons for this are unclear.

**Research design and methods:**

Prevalence and risk factors for diabetes at ages 40–69 years were compared in two population-based studies: Know Your Heart (KYH) (Russia, 2015–2018, n=4121) and the seventh wave of the Tromsø Study (Tromsø 7) (Norway, 2015–2016, n=17 649). Diabetes was defined by the level of glycated hemoglobin and/or self-reported diabetes and/or diabetes medication use. Marginal structural models were used to estimate the role of key risk factors for diabetes in differences between the studies.

**Results:**

Age-standardized prevalence of diabetes was higher in KYH compared with Tromsø 7 in men (11.6% vs 6.2%) and in women (13.2% vs 4.3%). Age-adjusted ORs for diabetes in KYH compared with Tromsø 7 were 2.01 (95% CI 1.68 to 2.40) for men and 3.66 (95% CI 3.13 to 4.26) for women. Adiposity (body mass index and waist circumference) explained none of this effect for men but explained 46.0% (39.6, 53.8) for women. Addition of smoking and C reactive protein, as further mediators, slightly increased the percentage explained of the difference between studies to 55.5% (46.5, 66.0) for women but only to 9.9% (−0.6, 20.8) for men.

**Conclusions:**

Adiposity is a key modifiable risk factor that appears to explain half of the almost threefold higher female prevalence of diabetes in Russia compared with Norway, but none of the twofold male difference.

**Significance of this study**

**What is already known about this subject?**

- Russia has strikingly higher diabetes rates compared with Western countries occurring within the context of a very high mortality from cardiovascular disease and low life expectancy at birth.
- Unlike many other countries diabetes is more common among women than men at older ages.

**What are the new findings?**

- Obesity and measures of adiposity contribute substantially to the higher prevalence of diabetes among women in Russia compared with women in Norway; however, obesity did not explain differences in diabetes prevalence among men between the two countries.
- Even after accounting for obesity and other risk factors, diabetes prevalence remained a lot higher in the Russian study than in Norway for both sexes.
- The proportion of undiagnosed diabetes in Russia is much higher than in Norway.

**How might these results change the focus of research or clinical practice?**

- Strategies to reduce levels of adiposity in the Russian population are important, with a need to focus on reducing levels in women.
- The proportion of undiagnosed diabetes in Russia should be reduced by targeted screening efforts.

## Introduction

Diabetes has an independent effect on the risk of cardiovascular events^1^ and causes long-term microvascular complications.^2 3^ The disease is heterogeneous in nature and progression and is broadly classified into type 1 diabetes and type 2 diabetes.^4^ Type 2 diabetes is strongly associated with obesity and related lifestyle factors and is the most common type in adults.^4^

Population aging and the worldwide rise in obesity have contributed to the marked rise in type 2 diabetes prevalence in many countries, although there remains substantial international variation.^5^ Data on type 2 diabetes prevalence in Russia have been reported in a few population-based studies based either on glycated hemoglobin^6–8^ or fasting glucose.^9–11^ These studies report relatively high prevalence of type 2 diabetes in Russia ranging from 7% to 16%, with the highest burden being in women compared with men at older ages. The notably higher prevalence in women compared with men is atypical compared with many other countries.^4 5^ The high prevalence of diabetes in Russia compared with the neighboring countries in Western Europe is of particular interest because this may contribute to the very high levels of cardiovascular disease (CVD) in Russia.^12^ However, no systematic attempt has been made previously to investigate which risk factors may explain the relatively high prevalence of diabetes in Russia compared with elsewhere.

At early stages symptoms of type 2 diabetes are absent or remain unnoticed; therefore a substantial proportion of type 2 diabetes remain undiagnosed and not managed.^13^ Previous studies in Russia reported a very high proportion of undiagnosed diabetes (up to 54%).^8^ This can lead to delay in management of the condition and healthcare interventions directed to reduce cardiometabolic risk factors such as hypercholesterolemia, hypertension, hypoglycemia, and obesity.

In this study we aim to investigate whether high diabetes prevalence in Russia compared with Norway is explained by known risk factors of diabetes. We used data collected in two recent cross-sectional, population-based studies conducted in Russia and Norway that defined diabetes in a comparable manner. These countries share a border and have similar population age structure.

## Methods

### Study populations

We used data on men and women aged 40–69 years who took part in two population-based studies. The Know Your Heart (KYH) study^14^ is a cross-sectional study conducted in Russia in 2015–2017. The seventh wave of Tromsø Study (Tromsø 7)^15^ was conducted among the residents of the municipality of Tromsø (Norway) in 2015–2016.

### Know Your Heart (Russia)

A random population-based sample of participants aged 35–69 years old (n=5071) stratified by age, sex and district were recruited in Arkhangelsk and Novosibirsk cities (Russia). Trained interviewers visited the sampled addresses and recorded information about residents’ health, sociodemographic characteristics and lifestyle (51% of approached agreed to participate). Participants were then invited to take part in a health check that usually occurred 1–2 weeks later in a research clinic and 4512 (35–69 years old) agreed (89%). Our analysis is based on 4121 participants aged 40–69 years who attended the health check (online supplemental figure 1). The health check included a medical examination, questionnaire and biological sample collection. The medical examination included blood pressure measurements and recording of weight and height. The questionnaire collected data on health problems, lifestyle, and medication use. The blood samples were non-fasting, but participants were asked not to eat and drink for 4 hours before the health check. Within 2 hours after venipuncture, blood samples were centrifuged, and serum was frozen at −80°C. Frozen samples were shipped to a laboratory in Moscow and analyzed in a single batch at the end of the fieldwork. Further details of the study design have been published elsewhere.^14^

10.1136/bmjdrc-2020-002021.supp7 Supplementary data

### Tromsø 7 (Norway)

All inhabitants of the municipality of Tromsø aged 40 years and above were invited to take part in Tromsø 7 and 21 083 participated (65%), of whom 17 649 aged 40–69 years were included in our analysis (online supplemental figure 2). All participants completed the questionnaires and examinations including biological sampling. The questionnaire included a broad set of questions on lifestyle, medication use, and disease. Blood samples (non-fasting) were processed immediately after collection and the laboratory assays of the biomarkers were done on the same day at the Department of Laboratory Medicine, University Hospital of Northern Norway (International Organization for Standardization (ISO) certification NS-EN ISO 15189:2012). Further details of the design of the Tromsø Study have been published elsewhere.^15^

10.1136/bmjdrc-2020-002021.supp8 Supplementary data

### Outcomes

The outcome of the study is the prevalence of diabetes mellitus, defined as glycated hemoglobin (HbA1c) >6.5% (48 mmol/mol)^16^ and/or self-reported diabetes and/or use of medication for diabetes (online supplemental table 1). Use of medication for diabetes was collected based on answers to the question about diabetes medication use and/or drugs taken during the last 4 weeks coded with A10 (antidiabetics) of the Anatomical Therapeutic Chemical classification.^17^ In KYH, diabetes self-report was determined from the question ‘Have you ever been told by a doctor or nurse that you have diabetes mellitus?’ In Tromsø 7, participants who answered that they currently have diabetes on the question ‘Have you or have you ever had diabetes?’ were recorded as having diabetes. A person with HbA1c ≥6.5% (48 mmol/mol) was considered undiagnosed if they did not report having diabetes or taking diabetes medications. Pre-diabetes was defined as HbA1c ≥5.7% (39 mmol/mol) and <6.5% (48 mmol/mol) among those who did not report that they have diabetes or take diabetes medications.^16^

10.1136/bmjdrc-2020-002021.supp1 Supplementary data

HbA1c was measured in whole blood by immunoturbidimetric test on AU680 Chemistry System (Beckman Coulter) in KYH and by capillary electrophoresis on Capillarys 3 Tera with the MCA Laboratory HbA1c calibrator traceable to the International Federation of Clinical Chemistry reference measurement in Tromsø 7. A calibration study between the two laboratories was conducted and HbA1c levels were adjusted appropriately to make them directly comparable.^18^

We were not able to distinguish between type 1 and type 2 diabetes in this study as consistent and comparable data on age at onset and other distinctive characteristics of these two conditions were not available.

### Exposure variables

Adiposity was assessed using body mass index (BMI) and waist circumference (WC). WC was measured at the narrowest part of the trunk (KYH) or at the umbilical level (Tromsø 7). To ensure comparability, WC in Tromsø 7 was converted to the narrowest waist using a conversion equation.^19^ Mean systolic and diastolic blood pressure were calculated as the mean of the second and third measurements. Smoking was categorized as ex-smokers, never-smokers and current smokers (1–10 cigarettes per day, 11–20 cigarettes per day, >20 cigarettes per day). Total cholesterol, low-density lipoprotein (LDL) cholesterol, high-density lipoprotein cholesterol, triglycerides, and high sensitivity C-reactive protein (hsCRP) were measured.^14 18^ A calibration study of the laboratory biomarkers was conducted to harmonize measurements between studies.^18^

All participants gave signed consent.

### Statistical analysis

Sex-specific prevalence of diabetes, pre-diabetes, and undiagnosed diabetes in the two studies were compared after age standardization to the Standard European Population 2013.

To examine if the differences in prevalence of diabetes between studies may be explained by different levels of diabetes risk factors, we conducted mediation analysis using marginal structural models,^20^ which allow the decomposition of total effect of exposure into that mediated by specific factors (indirect effect) and the remaining (direct) effect. In our analysis, the study (KYH vs Tromsø 7) was considered the exposure, while diabetes risk factors were considered possible mediators. BMI and WC were added together at the first step of the mediation analysis to provide an estimate of the role of adiposity, and the independent effects were not examined.^21^ Both BMI and WC were modeled as quintiles to account for non-linearity of the association by diabetes (online supplemental figures 3 and 4); quintiles were defined based on the total study population of men and women from both studies. A further model introduced smoking and hsCRP as additional mediators, the latter as it reflects proinflammatory status.^21 22^ Mediation analysis was done stratified by sex, and age was treated as a confounder.

10.1136/bmjdrc-2020-002021.supp9 Supplementary data

10.1136/bmjdrc-2020-002021.supp10 Supplementary data

Statistical analysis was performed using R V.3.6.0 (package medflex 0.66) and SAS V.9.4.

### Sensitivity analysis

Self-report of diabetes diagnosis may not be fully reliable. We therefore conducted a sensitivity analysis for mediation analyses and comparison of CVD risk factor profile where defined diabetes was based on HbA1c values alone.

## Results

Age-standardized prevalence of diabetes, pre-diabetes and undiagnosed diabetes was higher in KYH compared with Tromsø 7 among men and women (table 1 and online supplemental table 1). The proportion of those with diabetes who were undiagnosed was higher in KYH than in Tromsø 7 both in younger and older age groups (table 2).

**Table 1:** Number of people and age-standardized prevalence* of diabetes, undiagnosed diabetes, and pre-diabetes in Know Your Heart and Tromsø 7, by sex

	Know your Heart % (95% CI)†	Tromsø 7 % (95% CI)†
Men (n)	1732	8349
Diabetes cases (n)‡	219	514
Number of undiagnosed diabetes cases (n)§	76	94
Number of pre-diabetes cases (n)¶	612	1875
Diabetes mellitus prevalence‡	11.6 (10.3 to 12.8)	6.2 (5.6 to 6.7)
Undiagnosed diabetes§	4.04 (3.4 to 4.7)	1.15 (0.9 to 1.4)
Prevalence of pre-diabetes¶**	35.3 (33.2 to 37.4)	22.7 (21.8 to 23.6)
Women (n)	2389	9300
Diabetes cases (n)‡	361	395
Number of undiagnosed diabetes cases (n)§	91	48
Number of pre-diabetes cases (n)¶	824	2055
Diabetes mellitus prevalence‡	13.3 (12.3 to 14.2)	4.3 (3.8 to 4.8)
Undiagnosed diabetes§	3.5 (3.1 to 3.9)	0.5 (0.3 to 0.8)
Prevalence of pre-diabetes¶**	35.1 (33.4 to 36.9)	22.6 (21.7 to 23.4)

*Age-standardized to the Standard European Population 2013; p<0.001 for all comparisons.

†All percentages are based on the total number of participants in Know Your Heart and Tromsø 7.

‡Diabetes defined as HbA1c ≥6.5% (48 mmol/mol) and/or self-reported diabetes and/or use of medication with ATC code A10 (antidiabetics) according to the Anatomical Therapeutic Chemical (ATC) classification.

§Undiagnosed diabetes defined as HbA1c ≥6.5% (48 mmol/mol), no self-reported diabetes and no diabetes medication use.

¶Pre-diabetes defined as HbA1c ≥5.7% (39 mmol/mol) and <6.5% (48 mmol/mol), no self-reported diabetes and no diabetes medication use.

**Missing data: Know Your Heart: HbA1c 124, diabetes medication 423; Tromsø 7: self-report of diabetes 413; HbA1c 212.

HbA1c, glycated hemoglobin.

**Table 2:** Proportion of undiagnosed diabetes* among participants with measured HbA1c by sex and 15-year age groups in Tromsø 7 and Know Your Heart

	Know your Heart		Tromsø 7	
	Diabetes*† (n)	Undiagnosed diabetes*‡ % of diabetes cases (n)	Diabetes (n)	Undiagnosed diabetes % of diabetes cases (n)
Men				
Total sample	207	36.9 (76)	507	18.5 (94)
40–54 years old	55	38.3 (23)	191	19.9 (38)
55–69 years old	152	37.2 (58)	316	17.7 (56)
Women				
Total sample	342	26.8 (91)	389	12.3 (48)
40–54 years old	55	45.6 (26)	144	8.3 (12)
55–69 years old	287	23.7 (68)	245	14.7 (36)

*Number of diabetes and undiagnosed diabetes cases presented only for participants with complete data on HbA1c.

†Diabetes among participants with measured HbA1c was defined as HbA1c ≥6.5% (48 mmol/mol) and/or self-reported diabetes and/or use of medication with ATC code A10 (antidiabetics) according to the Anatomical Therapeutic Chemical (ATC) classification.

‡Undiagnosed diabetes among participants with measured HbA1c was defined as HbA1c ≥6.5% (48 mmol/mol), no self-reported diabetes and no diabetes medication use.

HbA1c, glycated hemoglobin.

Next we compared cardiometabolic risk factor profiles among participants with diabetes and without diabetes in both studies (table 3). In both studies men and women with diabetes had higher BMI and WC, higher systolic and diastolic blood pressure, and higher hsCRP levels than those who do not have diabetes. In KYH levels of total and LDL-cholesterol were similar in participants with and without diabetes; however, in Tromsø 7 total and LDL-cholesterol were lower in participants with diabetes. Smoking prevalence was similar in participants with and without diabetes in both studies. Use of lipid-lowering drugs was higher in Tromsø 7 study, in particular among participants with diabetes. Substantial differences in mean risk factor levels were observed between KYH and Tromsø 7. Systolic and diastolic blood pressure were higher in KYH than in Tromsø 7, and smoking prevalence was higher among men and mean BMI and WC were higher among women in KYH compared with Tromsø 7 (online supplemental table 6).

10.1136/bmjdrc-2020-002021.supp6 Supplementary data

**Table 3:** Cardiometabolic risk factors and smoking* in Know Your Heart and Tromsø 7, stratified by diabetes status and sex

	Know Your Heart Mean or proportion (95% CI)		Tromsø 7 Mean or proportion (95% CI)	
	With diabetes†	Without diabetes†	With diabetes†	Without diabetes†
Men (n)	219	1513	514	7835
BMI (mean, kg/m^2^)	32.1 (31.3 to 32.9)	27.2 (27.0 to 27.4)	30.4 (29.9 to 30.9)	27.8 (27.7 to 27.8)
Waist circumference (mean, cm)	109.0 (107.1 to 111.0)	95.4 (94.9 to 96.0)	105.7 (104.4 to 106.9)	97.9 (97.7 to 98.1)
Total cholesterol (mean, mmol/L)	5.34 (5.17 to 5.52)	5.27 (5.22 to 5.33)	4.97 (4.85 to 5.08)	5.49 (5.47 to 5.51)
HDL-cholesterol (mean, mmol/L)	1.17 (1.11 to 1.23)	1.35 (1.33 to 1.37)	1.23 (1.2 to 1.27)	1.38 (1.38 to 1.39)
LDL-cholesterol (mean, mmol/L)	3.50 (3.34 to 3.66)	3.46 (3.41 to 3.51)	3.16 (3.06 to 3.26)	3.73 (3.7 to 3.75)
Ln-transformed triglycerides (mean, mmol/L)	0.69 (0.6 to 0.78)	0.34 (0.32 to 0.37)	0.65 (0.59 to 0.71)	0.41 (0.4 to 0.42)
Ln-transformed hsCRP (mean, mmol/L)	1.00 (0.84 to 1.16)	0.29 (0.24 to 0.34)	0.48 (0.38 to 0.58)	0.04 (0.02 to 0.06)
SBP (mean, mm Hg)	142.1 (139.1 to 145.1)	137.3 (136.4 to 138.2)	135.7 (133.9 to 137.5)	130.7 (130.3 to 131.0)
DBP (mean, mm Hg)	87.1 (85.4 to 88.8)	86.4 (85.8 to 86.9)	78.9 (77.9 to 79.9)	78.8 (78.6 to 79.0)
Current smoker (proportion)	0.33 (0.26 to 0.4)	0.38 (0.36 to 0.41)	0.19 (0.16 to 0.23)	0.20 (0.19 to 0.21)
Use of lipid-lowering medications (ATC code C10) (proportion)	0.19 (0.14 to 0.24)	0.05 (0.04 to 0.07)	0.34 (0.3 to 0.38)	0.07 (0.07 to 0.08)
Women (n)	361	2028	395	8905
BMI (mean, kg/m^2^)	33.1 (32.3 to 33.9)	28.2 (28.0 to 28.4)	30.8 (30.0 to 31.6)	26.6 (26.5 to 26.7)
Waist circumference (mean, cm)	102.1 (100.3 to 103.9)	88.5 (88.0 to 89.0)	93.2 (91.5 to 94.9)	81.7 (81.5 to 82.0)
Total cholesterol (mean, mmol/L)	5.56 (5.39 to 5.73)	5.51 (5.46 to 5.55)	5.34 (5.18 to 5.5)	5.55 (5.52 to 5.57)
HDL-cholesterol (mean, mmol/L)	1.41 (1.35 to 1.46)	1.63 (1.61 to 1.65)	1.42 (1.37 to 1.47)	1.74 (1.73 to 1.75)
LDL-cholesterol (mean, mmol/L)	3.55 (3.4 to 3.7)	3.57 (3.53 to 3.61)	3.43 (3.29 to 3.57)	3.57 (3.55 to 3.59)
Ln-transformed triglycerides (mean, mmol/L)	0.65 (0.58 to 0.73)	0.22 (0.2 to 0.24)	0.63 (0.56 to 0.7)	0.14 (0.13 to 0.15)
Ln-transformed hsCRP (mean, mmol/L)	1.00 (0.86 to 1.14)	0.26 (0.21 to 0.3)	0.79 (0.66 to 0.91)	0.00 (−0.02 to 0.02)
SBP (mean, mm Hg)	137.7 (134.8 to 140.5)	127.6 (126.8 to 128.4)	135.6 (133.0 to 138.2)	123.2 (122.8 to 123.5)
DBP (mean, mm Hg)	83.2 (81.8 to 84.6)	80.9 (80.5 to 81.4)	74.3 (73.1 to 75.6)	72.6 (72.4 to 72.8)
Current smoker (proportion)	0.13 (0.09 to 0.18)	0.17 (0.15 to 0.19)	0.19 (0.15 to 0.25)	0.19 (0.18 to 0.2)
Use of lipid-lowering medications (ATC code C10) (proportion)	0.18 (0.14 to 0.22)	0.04 (0.03 to 0.04)	0.34 (0.29 to 0.39)	0.04 (0.04 to 0.05)

*Adjusted for age.

†Diabetes defined as HbA1c ≥6.5% (48 mmol/mol) and/or self-reported diabetes and/or use of medication with ATC code A10 (antidiabetics) according to the Anatomical Therapeutic Chemical (ATC) classification.

BMI, body mass index; DBP, diastolic blood pressure; HbA1c, glycated hemoglobin; HDL, high-density lipoprotein; hsCRP, high sensitivity C-reactive protein; LDL, low-density lipoprotein; SBP, systolic blood pressure.

The age-adjusted odds of having diabetes in KYH were twice that in Tromsø 7 among men and more than three times higher among women, as shown by the size of the total effect in table 4. Differences in distribution of BMI and WC did not appear to explain any of the differences in diabetes prevalence between study populations among men. The estimation of natural indirect effect of all mediators considered (BMI, WC, hsCRP, smoking) among men yielded an OR of 1.07 (0.99, 1.16), which indicates a small contribution of mediators to the differences in diabetes prevalence between studies (total effect). This is reflected by a small mediated percentage: 9.9% (−0.6, 20.8). On the contrary, the natural indirect effect of two measures of adiposity (BMI, WC) among women was estimated from an OR of 1.81 (1.68, 1.94), which corresponds to 46.0% (39.6, 53.8) of the diabetes differences between KYH and Tromsø 7 being explained by these two mediators. With addition of two remaining mediators (hsCRP and smoking), the natural indirect effect slightly increased to OR=2.04 (1.85, 2.26), which corresponds to 55.5% (46.5, 66.0) of the difference in prevalence between studies in women explained by all considered mediators (table 4). It was notable that the residual (natural direct effect) effects not mediated by BMI and WC were similar in men and women.

**Table 4:** OR* showing natural direct and indirect effects† of study (KYH vs Tromsø 7) on diabetes‡ prevalence assessed from mediation analyses and mediated percentage for different sets of risk factors (BMI, waist circumference, smoking, hsCRP), by sex

	Model 1 BMI and waist circumference included as mediators	Model 2 BMI, waist circumference, smoking, and hsCRP included as mediators
Men		
Natural direct effect	2.02 (1.70, 2.40)	1.87 (1.57, 2.24)
Natural indirect effect	0.99 (0.95, 1.04)	1.07 (0.99, 1.16)
Total effect	2.01 (1.68, 2.40)	2.01 (1.69, 2.38)
Percentage mediated	−1.1% (−8.9, 5.5)	9.9% (−0.6, 20.8)
Women		
Natural direct effect	1.99 (1.70, 2.35)	1.77 (1.49, 2.11)
Natural indirect effect	1.81 (1.68, 1.94)	2.04 (1.85, 2.26)
Total effect	3.66 (3.13, 4.26)	3.62 (3.10, 4.21)
Percentage mediated	46.0% (39.6, 53.8)	55.5% (46.5, 66.0)

*Adjusted for age.

†Total effect of exposure is decomposed into natural direct and indirect effects. Natural indirect effect means effect of exposure that is mediated by a specific set of risk factors. Natural direct effect is the remaining effect of an exposure after quantifying the natural indirect effect. In our analysis, the study (KYH vs Tromsø 7) was considered the exposure, while diabetes risk factors were considered possible mediators.

‡Diabetes defined as HbA1c ≥6.5% (48 mmol/mol) and/or self-reported diabetes and/or use of medication with ATC code A10 (antidiabetics) according to the Anatomical Therapeutic Chemical (ATC) classification.

BMI, body mass index; HbA1c, glycated hemoglobin; hsCRP, high sensitivity C reactive protein; KYH, Know Your Heart.

### Sensitivity analysis

The results of the mediation analysis were similar if conducted separately for diagnosed and undiagnosed diabetes (online supplemental tables 4 and 5); however, the contribution of BMI and WC to the mediation was smaller for undiagnosed than diagnosed diabetes.

10.1136/bmjdrc-2020-002021.supp4 Supplementary data

10.1136/bmjdrc-2020-002021.supp5 Supplementary data

Sensitivity analysis using a diabetes case definition based solely on HbA1c values did not substantially change the findings of the mediation analysis or of the comparison of the CVD risk factor profile (data available from authors).

## Discussion

In this study we compared the prevalence of diabetes in two population-based studies conducted in Russia and Norway using the same case definitions. We found much higher prevalence of diabetes in KYH (11.6% in men and 13.2% in women) compared with Tromsø 7 (6.2% in men and 4.3% in women). The prevalence of diabetes was higher in women than in men in the Russian sample, which is the opposite of what is observed in Norway and other countries.^4 5^ We also found that there is a higher proportion of undiagnosed diabetes in Russia than in Norway, with proportions of previously undiagnosed diabetes of 36.9% among men and 26.8% among women in KYH.

We attempted to explain the differences in prevalence of diabetes between the two countries using mediation analysis and found that adiposity measured by BMI and WC could explain up to 46% of the difference in diabetes prevalence between studies in women, but did not explain the differences between studies observed in men. Taking further account of smoking and hsCRP as mediation factors in addition to adiposity could explain 55.5% of the differences in diabetes prevalence between studies in women.

Our estimates of diabetes prevalence in Russia are in line with previous studies, although not all of them are published in the peer review literature or contain sufficient detail on age-specific diabetes prevalence.^7 10 11 23^ Two recent multiregion studies in Russia reported age-specific prevalence of diabetes and found that women at older ages have higher prevalence of diabetes than men.^8 9^ The NATION study (2013–2015) estimated type 2 diabetes prevalence based on both HbA1c and self-report: 7.0% of women vs 7.9% of men aged 45–59 years old and 14.1% of women vs 9.9% of men aged 60–79 years had diabetes.^8^ The ESSE-RF study (10 regions of the Russian Federation, 2012–2014) estimated the prevalence of diabetes mellitus based on self-report and fasting glucose: 9.4% of men and 7.4% of women aged 45–54 years old and 13.6% of men and 16.5% of women aged 55–64 years old had diabetes mellitus.^9^ Similarly to our study, other studies conducted in Russia report that a high proportion of diabetes is undiagnosed: 54% in NATION study,^8^ 43% in HAPIEE,^10^ and 27% in UEMS.^11^ Differences between these estimates and estimates from our study can be explained by the different age structures of the studied populations, different access to healthcare services in Russian regions, and different methods for diabetes prevalence estimates.

According to the WHO STEPwise approach to surveillance (STEPS) (2019), raised fasting blood glucose (≥7.0 mmol/L) or under medication for raised blood glucose was found in 7.1% of the Ukrainian population: 6.7% of men and 7.4% of women (18–69 years old). Nearly half of them had not previously been diagnosed with diabetes.^24^ The percentage of population with diabetes is lower in Belarus (3.2 of men and 3.9 of women) and higher in the Republic of Moldova (11.5 of men and 13.0 of women); however, in all three countries the prevalence of diabetes in women is higher than in men.^25^ The higher prevalence of diabetes in women than men observed in KYH is the opposite pattern to that observed in Norway and most other countries where the majority of the population are of European ancestry. The higher diabetes prevalence in men is usually explained by diverse biological, cultural, lifestyle, and environmental factors.^26–29^ Explanations for the pattern observed in Russia require further research. Certain cultural factors in Russia may be considered distal, that is, influencing behavior, and modify the biologically lower predisposition of women to develop diabetes.

Estimates of the prevalence of diabetes in Norway are available from national registries with prospectively collected data on prescriptions of antidiabetic drugs and diabetes diagnoses from hospitals and primary care visits for all residents in Norway aged 30–89 years. Crude prevalence of type 2 diabetes increased from 4.9% to 6.1% from 2009 to 2014, and diabetes prevalence was higher in men than in women (6.8% vs 5.3% in 2014).^30^ However, these estimates do not include undiagnosed diabetes cases that would be detected by screening. Intensive pharmacological and lifestyle management of diabetes delays onset and slows the progression of diabetes complications.^31 32^ Our study has shown that the proportion of undiagnosed diabetes is apparently smaller in the Norwegian study compared with the Russian study, but is still of significant public health concern given the potential health consequences of unmanaged diabetes.^33^

Weight reduction and diet modification interventions in people with impaired glucose tolerance reduced the incidence of diabetes in randomized controlled trials.^34 35^ Therefore, lifestyle interventions would be beneficial for both persons with clinically defined diabetes and persons with pre-diabetes.^36^ In our study prevalence of pre-diabetes was higher in KYH compared with Tromsø 7, which means there is much potential for diabetes prevention. Incorporation of a broader definition of pre-diabetes (HbA1c ≥5.7% or fasting glucose ≥5.6 mmol/L) to the diagnosis guidelines in Russia may be justified to prevent more cases of diabetes with timely intervention if the change in cut-points is shown to be cost-effective.

Our data do not explain in full why prevalence of diabetes differs in Norway and Russia particularly among men. Our measures of adiposity (BMI and WC) explained a substantial proportion of difference among women (46%), but these factors did not make an important contribution to differences among men. Interestingly, after accounting for adiposity the remaining difference in diabetes prevalence between KYH and Tromsø 7 study was similar for men and women (double the odds of diabetes prevalence). It was previously shown even among people of European ancestry that differences exist in the relationship between body fat and BMI.^37^ Also, the association of obesity and diabetes was shown to be stronger in low education groups, which suggests that socioeconomic circumstances may influence vulnerability to adiposity.^38^

It has been previously demonstrated that smoking is associated with diabetes, with a relative risk of 1.4 (adjusted for the baseline BMI).^22 39^ hsCRP reflects the level of general inflammation and is positively associated with obesity and diabetes.^21^ As the prevalence of smoking and hsCRP levels are higher among Russian men compared with men in Norway, we expected them to contribute to some of the difference in diabetes prevalence. However, we did not observe an additional contribution of these factors to explaining the differences in diabetes prevalence when adiposity measures were already included in the model. Among women, smoking and hsCRP made a small additional contribution to the difference in diabetes prevalence between studies after accounting for adiposity.

There are other potential explanations for the differences in diabetes prevalence between studies, such as diet,^40 41^ levels of physical activity,^42^ and sedentary behavior.^43^ Unfortunately, comparable data on these factors between our two studies are not available.

Type 2 diabetes is a multifactorial disease and involves genetic, behavioral and environmental factors, and their interaction.^44^ However, researchers still have a limited understanding of the genetic and epigenetic contribution to type 2 diabetes: only 10%–15% of heritability can be explained by known genetic variants.^45^ At the present time we do not have genomic data for both studies in order to investigate any differences between them.

### Limitations

The major limitation of the mediation analysis in our study is the cross-sectional nature of the data. People who knew they had diabetes could have attempted to lose weight, increase physical activity, eat a healthier diet, and stop smoking. For example, lower LDL-cholesterol levels in participants with diabetes in Tromsø 7 study can in part be explained by higher use of lipid-lowering medications and changes in diet after diabetes diagnosis. Beyond this it is likely that our anthropometric measures of adiposity in the two populations failed to adequately capture differences in the extent of visceral abdominal adiposity, which is particularly strongly related to risk of diabetes.^46^ Validity of the mediation estimates is dependent on the assumption of no uncontrolled confounding for the exposure–outcome, exposure–mediator, or mediator-outcome relations.^20^

Although we were not able to distinguish between type 1 diabetes and type 2 diabetes in our study, our results will be principally driven by type 2 diabetes because it constitutes between 90% and 95% of all diabetes in these populations.^4^

Finally, care must be taken before generalizing the study findings to the populations of Russia and Norway as a whole. First, the studies were conducted in three cities whose characteristics will differ in some respects from the national populations. In addition there is the uncertainty about whether the participants we studied were representative of their own cities’ populations. The Tromsø 7 study had a good response rate (65%), as did the study in Arkhangelsk (68%), although in Novosibirsk the response rate was low (41%).^14^ The participants who did not attend the health check in KYH study were likely to have more adverse risk factor profile than those who did (online supplemental tables 2 and 3). However it is notable that our estimates of diabetes prevalence from KYH are consistent with those of other population-based studies in Russia. Similarly, prevalence estimates for diabetes in Tromsø 7 are similar to the study reporting diabetes prevalence in the whole of Norway. Potential explanatory (mediating) effects of specific biomedical markers are likely not to be affected.

10.1136/bmjdrc-2020-002021.supp2 Supplementary data

10.1136/bmjdrc-2020-002021.supp3 Supplementary data

## Conclusions

The major differences in diabetes prevalence between Russia and Norway have important implications for health services in Russia and could contribute to the differences in CVD mortality between the two countries. Adiposity indices, smoking and C reactive protein only partially explained the differences in diabetes prevalence between studies in women and did not explain differences between diabetes prevalence in men. A substantial proportion of unexplained differences remained and requires further investigation. People with undiagnosed diabetes are not prescribed recommended glucose-lowering, blood pressure-lowering and lipid-lowering drugs, as well as antismoking counseling, which can be expected to reduce the risk of CVD and other complications of diabetes. The proportion of undiagnosed diabetes in Russia is alarmingly high given potential health consequences for individuals and subsequent burden from avoidable complications of diabetes on the healthcare system.

## References

[1] Prospective Studies Collaboration and Asia Pacific Cohort Studies Collaboration. Sex-specific relevance of diabetes to occlusive vascular and other mortality: a collaborative meta-analysis of individual data from 980 793 adults from 68 prospective studies. Lancet Diabetes Endocrinol 2018;6:538–46. 10.1016/S2213-8587(18)30079-2 29752194 PMC6008496

[2] Selvin E, Ning Y, Steffes MW, et al. Glycated hemoglobin and the risk of kidney disease and retinopathy in adults with and without diabetes. Diabetes 2011;60:298–305. 10.2337/db10-1198 20978092 PMC3012185

[3] Colagiuri S, Lee CMY, Wong TY, et al. Glycemic thresholds for diabetes-specific retinopathy: implications for diagnostic criteria for diabetes. Diabetes Care 2011;34:145–50. 10.2337/dc10-1206 20978099 PMC3005450

[4] Skyler JS, Bakris GL, Bonifacio E, et al. Differentiation of diabetes by pathophysiology, natural history, and prognosis. Diabetes 2017;66:241–55. 10.2337/db16-0806 27980006 PMC5384660

[5] NCD Risk Factor Collaboration (NCD-RisC). Worldwide trends in diabetes since 1980: a pooled analysis of 751 population-based studies with 4.4 million participants. Lancet 2016;387:1513–30. 10.1016/S0140-6736(16)00618-8 27061677 PMC5081106

[6] Metelskaya VA, Shalnova SA, Deev AD, et al. Analysis of atherogenic dyslipidemias prevalence among population of Russian Federation (results of the ESSE-RF study). Profil med 2016;19:15. 10.17116/profmed201619115-23

[7] Oksuzyan A, Shkolnikova M, Vaupel JW, et al. Sex differences in biological markers of health in the study of stress, aging and health in Russia. PLoS One 2015;10:e0131691. 10.1371/journal.pone.0131691 26121035 PMC4484801

[8] Dedov I, Shestakova M, Benedetti MM, et al. Prevalence of type 2 diabetes mellitus (T2DM) in the adult Russian population (nation study). Diabetes Res Clin Pract 2016;115:90–5. 10.1016/j.diabres.2016.02.010 27107818

[9] Zhernakova YV, Chazova IE, Oshchepkova EV, et al. The prevalence of diabetes mellitus in population of hypertensive patients according to ESSE rf study results. Systemic Hypertension 2018;15:56–62. 10.26442/2075-082X_15.1.56-62

[10] Mustafina SV, Rymar OD, Malyutina SK, et al. Prevalence of diabetes in the adult population of Novosibirsk. Diabetes mellitus 2017;20:329–34. 10.14341/DM8744

[11] Bikbov MM, Fayzrakhmanov RR, Kazakbaeva GM, et al. Prevalence, awareness and control of diabetes in Russia: the Ural eye and medical study on adults aged 40+ years. PLoS One 2019;14:e0215636. 10.1371/journal.pone.0215636 31009496 PMC6476495

[12] Townsend N, Wilson L, Bhatnagar P, et al. Cardiovascular disease in Europe: epidemiological update 2016. Eur Heart J 2016;37:3232–45. 10.1093/eurheartj/ehw334 27523477 PMC13480964

[13] Cowie CC, Rust KF, Byrd-Holt DD, et al. Prevalence of diabetes and high risk for diabetes using A1c criteria in the U.S. population in 1988-2006. Diabetes Care 2010;33:562–8. 10.2337/dc09-1524 20067953 PMC2827508

[14] Cook S, Malyutina S, Kudryavtsev AV, et al. Know your heart: rationale, design and conduct of a cross-sectional study of cardiovascular structure, function and risk factors in 4500 men and women aged 35-69 years from two Russian cities, 2015-18. Wellcome Open Res 2018;3:67. 10.12688/wellcomeopenres.14619.1 30123849 PMC6073094

[15] Jacobsen BK, Eggen AE, Mathiesen EB, et al. Cohort profile: the Tromso study. Int J Epidemiol 2012;41:961–7. 10.1093/ije/dyr049 21422063 PMC3429870

[16] American Diabetes Association. 2. Classification and Diagnosis of Diabetes: Standards of Medical Care in Diabetes-2019. Diabetes Care 2019;42:S13–28. 10.2337/dc19-S002 30559228

[17] Carr-Hill R. The measurement of inequities in health: lessons from the British experience. Soc Sci Med 1990;31:393–404. 10.1016/0277-9536(90)90286-2 2218619

[18] Lakunchykova O, Averina M, Wilsgaard T, et al. Why does Russia have such high cardiovascular mortality rates? comparisons of blood-based biomarkers with Norway implicate non-ischaemic cardiac damage. J Epidemiol Community Health 2020;74:698–704. 10.1136/jech-2020-213885 32414935 PMC7577103

[19] Mason C, Katzmarzyk PT. Variability in waist circumference measurements according to anatomic measurement site. Obesity 2009;17:1789–95. 10.1038/oby.2009.87 19343017

[20] Lange T, Vansteelandt S, Bekaert M. A simple unified approach for estimating natural direct and indirect effects. Am J Epidemiol 2012;176:190–5. 10.1093/aje/kwr525 22781427

[21] Kahn SE, Cooper ME, Del Prato S. Pathophysiology and treatment of type 2 diabetes: perspectives on the past, present, and future. Lancet 2014;383:1068–83. 10.1016/S0140-6736(13)62154-6 24315620 PMC4226760

[22] Pan A, Wang Y, Talaei M, et al. Relation of active, passive, and quitting smoking with incident type 2 diabetes: a systematic review and meta-analysis. Lancet Diabetes Endocrinol 2015;3:958–67. 10.1016/S2213-8587(15)00316-2 26388413 PMC4656094

[23] Metelskaya VA, Shkolnikova MA, Shalnova SA, et al. Prevalence, components, and correlates of metabolic syndrome (Mets) among elderly Muscovites. Arch Gerontol Geriatr 2012;55:231–7. 10.1016/j.archger.2011.09.005 21955584 PMC3276749

[24] World Health Organization. Risk factors for noncommunicable diseases in Ukraine in 2019. Summary of results from the who steps survey and comparison with selected countries, 2020. Available: https://www.euro.who.int/__data/assets/pdf_file/0003/469173/Risk-factors-NCD-Ukraine-summary-eng.pdf

[25] World Health Organization Gender and non-communicable disease in Europe. Analysis of steps data 2020, 2020. Available: https://apps.who.int/iris/bitstream/handle/10665/337471/WHO-EURO-2020-1664-41415-56457-eng.pdf

[26] Tramunt B, Smati S, Grandgeorge N, et al. Sex differences in metabolic regulation and diabetes susceptibility. Diabetologia 2020;63:453–61. 10.1007/s00125-019-05040-3 31754750 PMC6997275

[27] Kautzky-Willer A, Harreiter J, Pacini G. Sex and gender differences in risk, pathophysiology and complications of type 2 diabetes mellitus. Endocr Rev 2016;37:278–316. 10.1210/er.2015-1137 27159875 PMC4890267

[28] Lagou V, Mägi R, Hottenga J-J, et al. Sex-dimorphic genetic effects and novel loci for fasting glucose and insulin variability. Nat Commun 2021;12:24. 10.1038/s41467-020-19366-9 33402679 PMC7785747

[29] Morris AP, Voight BF, Teslovich TM, et al. Large-Scale association analysis provides insights into the genetic architecture and pathophysiology of type 2 diabetes. Nat Genet 2012;44:981–90. 10.1038/ng.2383 22885922 PMC3442244

[30] Ruiz PLD, Stene LC, Bakken IJ, et al. Decreasing incidence of pharmacologically and non-pharmacologically treated type 2 diabetes in Norway: a nationwide study. Diabetologia 2018;61:2310–8. 10.1007/s00125-018-4681-4 29995214 PMC6182655

[31] Diabetes Control and Complications Trial Research Group, Nathan DM, Genuth S, et al. The effect of intensive treatment of diabetes on the development and progression of long-term complications in insulin-dependent diabetes mellitus. N Engl J Med 1993;329:977–86. 10.1056/NEJM199309303291401 8366922

[32] Gæde P, Vedel P, Larsen N, et al. Multifactorial intervention and cardiovascular disease in patients with type 2 diabetes. N Engl J Med Overseas Ed 2003;348:383–93. 10.1056/NEJMoa021778 12556541

[33] Langholz PL, Wilsgaard T, Njølstad I. Trends in known and undiagnosed diabetes, HbA1c levels, cardio-metabolic risk factors and diabetes treatment target achievement in repeated cross-sectional surveys – the Tromsø study 1994-2016. medRxiv 2020. 10.1101/2020.10.30.20222117 PMC7993331 33757943

[34] Lindström J, Ilanne-Parikka P, Peltonen M, et al. Sustained reduction in the incidence of type 2 diabetes by lifestyle intervention: follow-up of the Finnish diabetes prevention study. Lancet 2006;368:1673–9. 10.1016/S0140-6736(06)69701-8 17098085

[35] Knowler WC, Barrett-Connor E, Fowler SE, et al. Reduction in the incidence of type 2 diabetes with lifestyle intervention or metformin. N Engl J Med 2002;346:393–403. 10.1056/NEJMoa012512 11832527 PMC1370926

[36] American Diabetes Association. 3. Prevention or delay of type 2 diabetes: standards of medical care in diabetes-2019. Diabetes Care 2019;42:S29–33. 10.2337/dc19-S003 30559229

[37] Deurenberg P, Yap M, van Staveren WA. Body mass index and percent body fat: a meta analysis among different ethnic groups. Int J Obes Relat Metab Disord 1998;22:1164–71. 10.1038/sj.ijo.0800741 9877251

[38] Diderichsen F, Andersen I. The syndemics of diabetes and depression in Brazil - an epidemiological analysis. SSM Popul Health 2019;7:100318–2. 10.1016/j.ssmph.2018.11.002 PMC6293027 30581954

[39] Willi C, Bodenmann P, Ghali WA, et al. Active smoking and the risk of type 2 diabetes: a systematic review and meta-analysis. JAMA 2007;298:2654–64. 10.1001/jama.298.22.2654 18073361

[40] Meyer KA, Kushi LH, Jacobs DR, et al. Carbohydrates, dietary fiber, and incident type 2 diabetes in older women. Am J Clin Nutr 2000;71:921–30. 10.1093/ajcn/71.4.921 10731498

[41] Esposito K, Kastorini C-M, Panagiotakos DB, et al. Prevention of type 2 diabetes by dietary patterns: a systematic review of prospective studies and meta-analysis. Metab Syndr Relat Disord 2010;8:471–6. 10.1089/met.2010.0009 20958207

[42] Aune D, Norat T, Leitzmann M, et al. Physical activity and the risk of type 2 diabetes: a systematic review and dose-response meta-analysis. Eur J Epidemiol 2015;30:529–42. 10.1007/s10654-015-0056-z 26092138

[43] Patterson R, McNamara E, Tainio M, et al. Sedentary behaviour and risk of all-cause, cardiovascular and cancer mortality, and incident type 2 diabetes: a systematic review and dose response meta-analysis. Eur J Epidemiol 2018;33:811–29. 10.1007/s10654-018-0380-1 29589226 PMC6133005

[44] Langenberg C, Lotta LA. Genomic insights into the causes of type 2 diabetes. The Lancet 2018;391:2463–74. 10.1016/S0140-6736(18)31132-2 29916387

[45] Kwak SH, Park KS. Recent progress in genetic and epigenetic research on type 2 diabetes. Exp Mol Med 2016;48:e220. 10.1038/emm.2016.7 26964836 PMC4892885

[46] Boyko EJ, Fujimoto WY, Leonetti DL, et al. Visceral adiposity and risk of type 2 diabetes: a prospective study among Japanese Americans. Diabetes Care 2000;23:465–71. 10.2337/diacare.23.4.465 10857936

