# Supplementary figures and images for: What factors explain the much higher diabetes prevalence in Russia compared with Norway? Major sex differences in the contribution of adiposity
Source: BMJ Open Diabetes Res Care. 2021 Mar 4;9(1):e002021. doi: 10.1136/bmjdrc-2020-002021 (PMC7934764; doi:10.1136/bmjdrc-2020-002021)

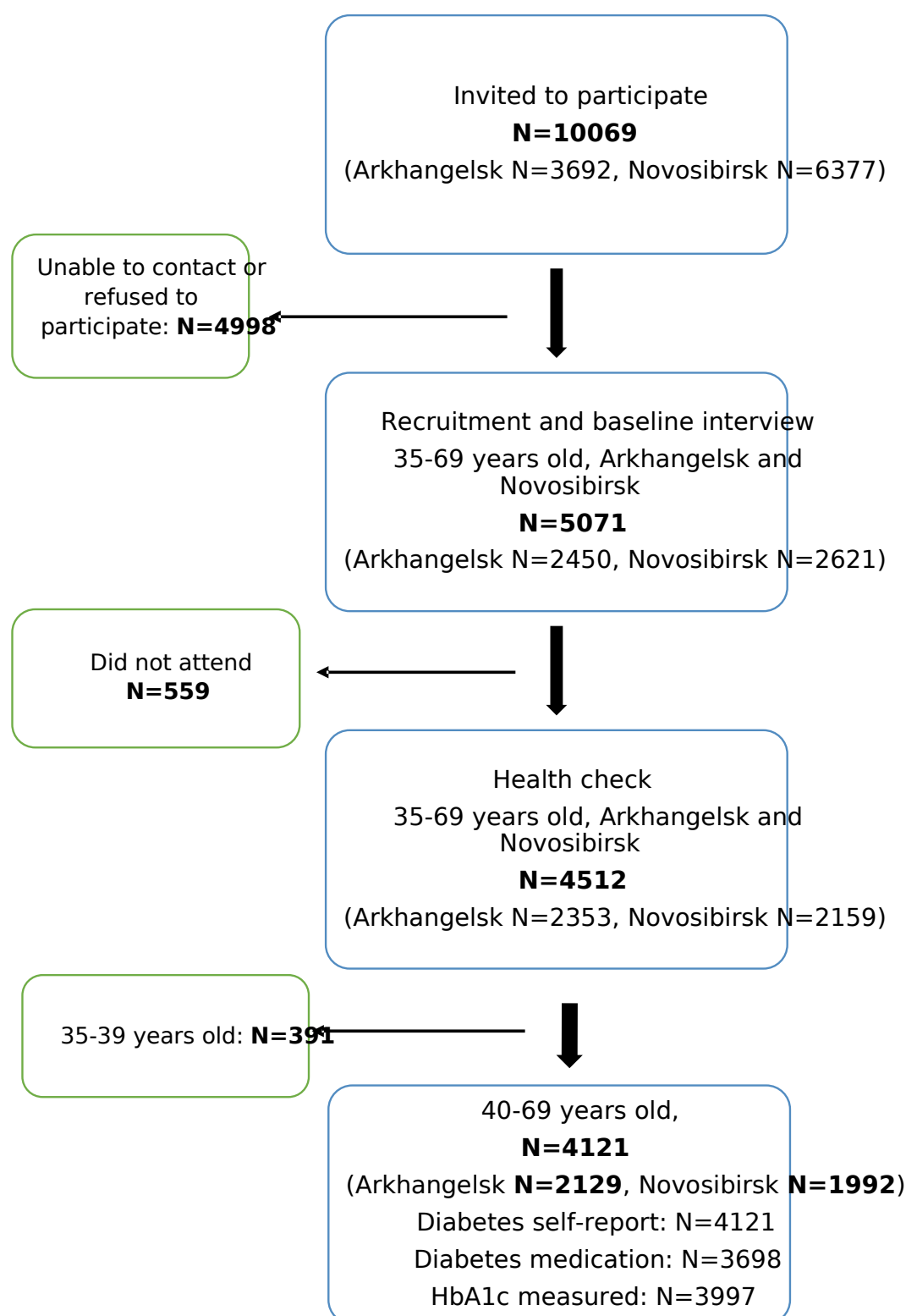

Supplementary Figure 1: Flow chart for Know Your Heart study

Supplement: Supplementary data [file bmjdrc-2020-002021supp007.pdf]

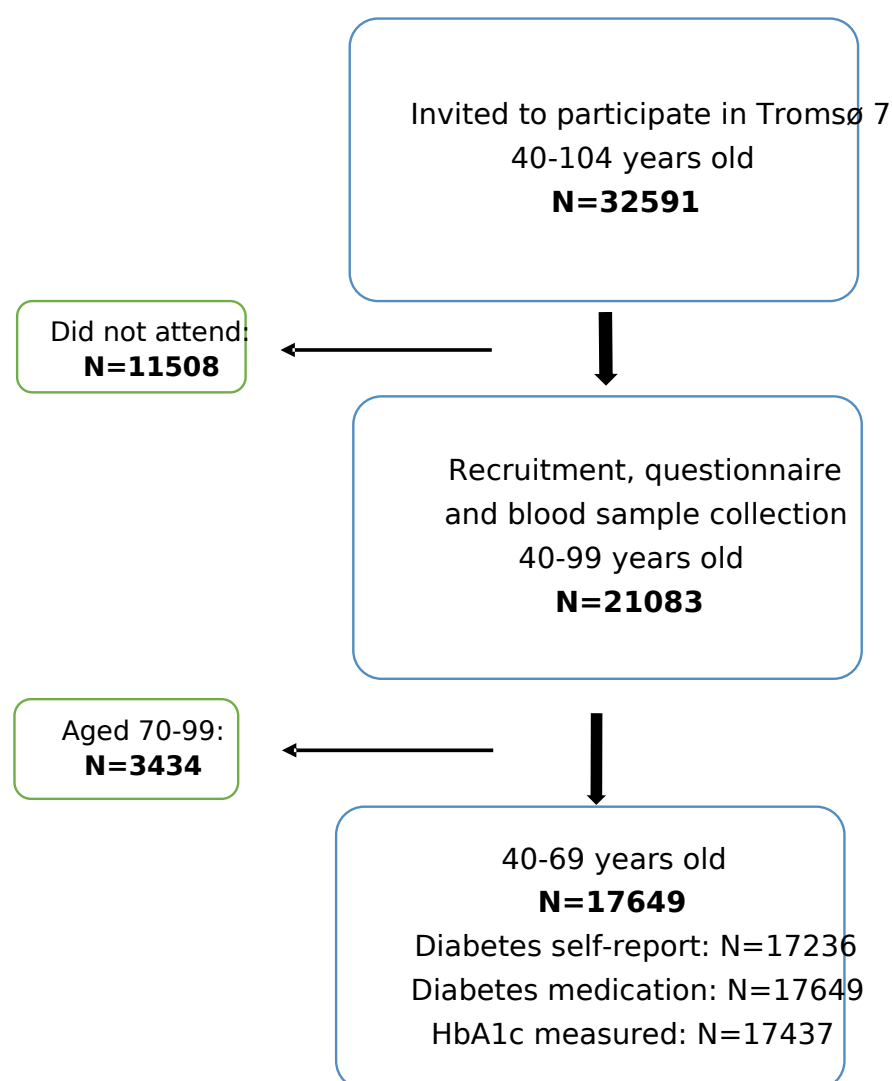

Supplementary Figure 2. Flow chart for Tromsø 7

Supplement: Supplementary data [file bmjdrc-2020-002021supp008.pdf]
