# Supplementary material for: What factors explain the much higher diabetes prevalence in Russia compared with Norway? Major sex differences in the contribution of adiposity
Source: BMJ Open Diabetes Res Care. 2021 Mar 4;9(1):e002021. doi: 10.1136/bmjdrc-2020-002021 (PMC7934764; doi:10.1136/bmjdrc-2020-002021)
Supplement: Supplementary data [file bmjdrc-2020-002021supp001.pdf]

Supplementary Table 1. Number of cases of diabetes mellitus in KYH and Tromsø 7 according to different criteria of case definition.

	KYH (%, N)[a]	Tromsø 7 (%, N)[a]
Men		
HbA1c ≥ 6.5% (48 mmol/mol)	76.7 (168)	78.6 (404)
Self-report of diabetes	64.4 (141)	76.5 (393)
Use of medication for diabetes	41.6 (91)	68.5 (352)
Any of the above (total diabetes cases)	219	514
Women		
HbA1c ≥ 6.5% (48 mmol/mol)	63.4 (229)	66.8 (264)
Self-report of diabetes	70.6 (255)	78.7 (311)
Use of medication for diabetes	52.3 (189)	68.9 (272)
Any of the above (total diabetes cases)	361	395

[a] Percent of total diabetes cases
