# Supplementary material for: What factors explain the much higher diabetes prevalence in Russia compared with Norway? Major sex differences in the contribution of adiposity
Source: BMJ Open Diabetes Res Care. 2021 Mar 4;9(1):e002021. doi: 10.1136/bmjdrc-2020-002021 (PMC7934764; doi:10.1136/bmjdrc-2020-002021)
Supplement: Supplementary data [file bmjdrc-2020-002021supp006.pdf]

Supplementary Table 6. Cardiometabolic risk factors [a] in Know Your Heart and Tromsø 7 stratified by sex.

	Mean or proportion (95% CI)	
	KYH	Tromsø 7
**Men (N)**	1722	8349
BMI (mean, kg/m2)	27.7 (27.5, 27.9)	27.9 (27.8, 28.0)
Waist circumference (mean, cm)	96.9 (96.4, 97.4)	98.4 (98.2, 98.6)
Total cholesterol (mean, mmol/L)	5.27 (5.22, 5.32)	5.46 (5.43, 5.48)
HDL-cholesterol (mean, mmol/L)	1.33 (1.31, 1.35)	1.37 (1.37, 1.38)
LDL- cholesterol (mean, mmol/L)	3.45 (3.4, 3.5)	3.69 (3.67, 3.71)
Ln-transformed triglycerides, (mean, mmol/L)	0.38 (0.35, 0.41)	0.43 (0.41, 0.44)
Ln-transformed CRP, (mean, mmol/L)	0.37 (0.32, 0.41)	0.06 (0.04, 0.08)
SBP (mean, mmHg)	137.6 (136.8, 138.5)	131.0 (130.6, 131.4)
DBP (mean, mmHg)	86.4 (85.9, 86.9)	78.8 (78.6, 79.0)
Current smoker (proportion)	0.37 (0.35, 0.39)	0.2 (0.19, 0.2)
Use of lipid lowering medications, (ATC code C10) (proportion)	0.07 (0.06, 0.08)	0.09 (0.08, 0.1)
**Women (N)**	2377	9300
BMI (mean, kg/m2)	28.8 (28.6, 29.1)	26.8 (26.7, 26.9)
Waist circumference (mean, cm)	90.3 (89.8, 90.7)	82.3 (82.0, 82.5)
Total cholesterol (mean, mmol/L)	5.47 (5.43, 5.51)	5.54 (5.52, 5.56)
HDL-cholesterol (mean, mmol/L)	1.60 (1.58, 1.62)	1.72 (1.71, 1.73)
LDL- cholesterol (mean, mmol/L)	3.53 (3.5, 3.57)	3.56 (3.55, 3.58)
Ln-transformed triglycerides, (mean, mmol/L)	0.26 (0.24, 0.28)	0.17 (0.16, 0.18)
Ln-transformed CRP, (mean, mmol/L)	0.33 (0.29, 0.37)	0.03 (0.01, 0.05)
SBP (mean, mmHg)	128.5 (127.7, 129.2)	123.8 (123.4, 124.1)
DBP (mean, mmHg)	81.1 (80.7, 81.5)	72.7 (72.5, 73.0)
Current smoker (proportion)	0.16 (0.15, 0.18)	0.19 (0.18, 0.2)
Use of lipid lowering medications, (ATC code C10) (proportion)	0.05 (0.04, 0.06)	0.05 (0.05, 0.06)

[a] Adjusted for age

[b] Diabetes defined as HbA1C ≥ 6.5% and/or self-reported diabetes and/or use of medication with ATC-code A10 (antidiabetics) according to the Anatomical Therapeutic Chemical (ATC) classification
