# Supplementary material for: What factors explain the much higher diabetes prevalence in Russia compared with Norway? Major sex differences in the contribution of adiposity
Source: BMJ Open Diabetes Res Care. 2021 Mar 4;9(1):e002021. doi: 10.1136/bmjdrc-2020-002021 (PMC7934764; doi:10.1136/bmjdrc-2020-002021)
Supplement: Supplementary data [file bmjdrc-2020-002021supp005.pdf]

Supplementary Table 5. Odds ratios [a] showing natural direct and indirect effects [c] of study (KYH vs Tromsø 7) on ***undiagnosed diabetes*** [b] prevalence assessed from mediation analyses and mediated percentage for different sets of risk factors (BMI, waist circumference, smoking, hsCRP) by sex

	Model 1<br>BMI and waist circumference included as mediators	Model 2<br>BMI, waist circumference, smoking, hsCRP included as mediators
Men		
Natural direct effect	4.08 (2.97, 5.60)	3.60 (2.58, 5.00)
Natural indirect effect	0.95 (0.89, 1.02)	1.08 (0.95, 1.22)
Total effect	3.89 (2.82, 5.35)	3.88 (2.84, 5.31)
Percentage mediated	-3.5% (-9.1, 1.3)	5.7% (-3.5, 15.3)
Women		
Natural direct effect	4.54 (2.98, 6.92)	4.33 (2.75, 6.81)
Natural indirect effect	1.63 (1.40, 1.89)	1.71 (1.36, 2.14)
Total effect	7.39 (5.09, 10.72)	7.38 (5.06, 10.75)
Percentage mediated	24.3% (15.8, 35.0)	26.7% (14.7,41.0)

[a] Adjusted for age

[b] Undiagnosed diabetes among participants with measured HbA1c was defined as HbA1c ≥ 6.5% (48 mmol/mol), no self-reported diabetes and no diabetes medication use

[c] Total effect of exposure is decomposed into natural direct and indirect effect. Natural indirect effect means effect of exposure that is mediated by specific set of risk factors. Natural direct effect is the remaining effect of an exposure after quantifying the natural indirect effect. In our analysis, the study (KYH vs Tromsø 7) was considered the exposure, while diabetes risk factors were considered possible mediators.
