# Supplementary material for: What factors explain the much higher diabetes prevalence in Russia compared with Norway? Major sex differences in the contribution of adiposity
Source: BMJ Open Diabetes Res Care. 2021 Mar 4;9(1):e002021. doi: 10.1136/bmjdrc-2020-002021 (PMC7934764; doi:10.1136/bmjdrc-2020-002021)
Supplement: Supplementary data [file bmjdrc-2020-002021supp002.pdf]

Supplementary Table 2. Differences in main study variables for KYH participants (general population sample) who attended the health check and who did not, women* (N=2619).

| | Attended the health check (N=2385) | Did not attend the health check (N=234) | P-value |
|---|---|---|---|
| Age (years), mean (sd), min-max | 55.9 (8.7)<br>40-69 | 54.7 (9.0)<br>40-69 | 0.047 |
| City | | | <0.0001 |
| Arkhangelsk, % (N) | 96.3 (1237) | 3.7 (48) | |
| Novosibirsk, % (N) | 86.1 (1148) | 13.9 (186) | |
| | | | |
| Married, % (N) | 51.1 (1218) | 41.9 (99) | 0.007 |
| Education less than college level, % (N) | 21.3 ( 519) | 31.2 (72) | 0.001 |
| In regular paid work, % (N) | 58.5 (1325) | 49.6 (124) | 0.028 |
| Not enough money for food or clothes, % (N) | 22.2 (534) | 26.4 (59) | 0.154 |
| Depression severity (PHQ-9)† ≥ 5, % (N) | 41.3 (986) | 37.4 (87) | 0.251 |
| Anxiety severity (GAD-7)† ≥ 5, % (N) | 26.8 (640) | 23.0 (54) | 0.21 |
| Drinker, % (N) | 81.9 (1936) | 72.4 (170) | 0.001 |
| CAGE† score total ≥ 2, % (N) | 3.7 (101) | 7.1 (20) | 0.008 |
| Current smoker, % (N) | 15.1 (383) | 24.7 (63) | <0.0001 |
| Blood pressure medication, % (N) | 36.1 (917) | 31.9 (76) | 0.239 |
| Lipid lowering medication, % (N) | 14.7 (433) | 10.7 (30) | 0.092 |
| Self-reported hypertension, % (N) | 53.4 (1269) | 52.9 (117) | 0.904 |
| Self-reported myocardial infarction, % (N) | 3.2 (113) | 2.7 (9) | 0.682 |
| Self-reported heart failure, % (N) | 15.0 (395) | 10.9 (27) | 0.087 |
| Self-reported stroke, % (N) | 2.8 (81) | 4.5 (12) | 0.116 |
| Self-reported diabetes, % (N) | 7.6 (244) | 8.7 (25) | 0.547 |
| Visited general practitioner more than once in the last 12 month, % (N) | 39.1 (942) | 28.2 (65) | 0.001 |
| Was hospitalized at least once in the last 12 month, % (N) | 15.1 (366) | 14.6 (34) | 0.848 |

*adjusted for age
†PHQ-9: Patient Health Questionnaire – 9; GAD-7: General Anxiety Disorder – 7; CAGE: “cut-annoyed-guilty-eye”, screening tool for alcohol-related problems.
