# Supplementary material for: What factors explain the much higher diabetes prevalence in Russia compared with Norway? Major sex differences in the contribution of adiposity
Source: BMJ Open Diabetes Res Care. 2021 Mar 4;9(1):e002021. doi: 10.1136/bmjdrc-2020-002021 (PMC7934764; doi:10.1136/bmjdrc-2020-002021)
Supplement: Supplementary data [file bmjdrc-2020-002021supp003.pdf]

Supplementary Table 3. Differences in main study variables for KYH participants who attended the health check and who did not, men* (N=1982).

	Attended the health check (N=1731)	Did not attend the health check (N=251)	P-value
Age (years), mean (sd), min-max	56.1 (8.5)<br>40-69	56.5 (8.7)<br>40-69	0.513
City			<0.0001
Arkhangelsk, % (N)	95.9 (888)	4.1 (38)	
Novosibirsk, % (N)	79.8 (843)	20.2 (213)	
Married, % (N)	78.3 (1351)	67.7 (170)	<0.0001
Education less than college level, % (N)	29.8 (519)	47.9 (121)	<0.0001
In regular paid work, % (N)	61.6 (1027)	49.7 (122)	0.001
Not enough money for food or clothes, % (N)	17.4 (301)	26.2 (66)	0.001
Depression severity (PHQ-9*)>=10, % (N)	26.0 (451)	28.6 (72)	0.387
Anxiety severity (GAD-7)>=5, % (N)	14.8 (257)	15.2 (38)	0.893
Drinker, % (N)	84.6 (1459)	79.5 (198)	0.037
CAGE score total>=2, % (N)	20.1 (352)	31.9 (80)	<0.0001
Current smoker, % (N)	36.3 (631)	62.0 (155)	<0.0001
Blood pressure medication, % (N)	31.4 (567)	18.3 (51)	<0.0001
Lipid lowering medication, % (N)	11.6 (218)	6.3 (18)	0.01
Self-reported Hypertension, % (N)	49.7 (860)	35.6 (92)	<0.0001
Self-reported Myocardial Infarction, % (N)	7.7 (156)	6.6 (20)	0.493
Self-reported heart failure, % (N)	9.9 (205)	11.0 (34)	0.549
Self-reported stroke (self-report), % (N)	3.7 (83)	5.1 (17)	0.231
Self-reported diabetes, % (N)	7.2 (134)	5.1 (14)	0.196
Visited general practitioner more than once in the last 12 month, % (N)	26.4 (465)	16.4 (43)	0.001
Was hospitalized at least once in the last 12 month, % (N)	16.1 (289)	14.8 (39)	0.577

*adjusted for age
†PHQ-9: Patient Health Questionnaire – 9; GAD-7: General Anxiety Disorder – 7; CAGE: “cut-annoyed-guilty-eye”, screening tool for alcohol-related problems.
